# Acute Granulomatous Thyroiditis With Abscess Formation Due To Brucellosis: A Case Report

**DOI:** 10.1155/crie/9644135

**Published:** 2025-09-08

**Authors:** Soroosh Moradi Dastjerdi, Farnaz Karimi Ghahderijani, Raheleh Sadat Sajad

**Affiliations:** Isfahan Endocrine and Metabolism Research Center, Isfahan University of Medical Sciences, Isfahan, Iran

**Keywords:** abscess, brucellosis, case reports, suppurative, thyroiditis

## Abstract

This case report discusses a rare instance of acute granulomatous thyroiditis resulting from brucellosis. A 47-year-old female cattle farmer presented with painful neck swelling and systemic symptoms. Initial investigations suggested a potential malignancy, but further testing, such as serological tests, pathology and imaging revealed brucellosis as the underlying cause. The patient underwent surgical intervention for abscess drainage and antibiotic therapy for brucellosis and showed significant clinical improvement. This case underscores the need for awareness of *Brucella melitensis*, a zoonotic infection, as a differential diagnosis in thyroid conditions, contributing to the understanding of its varied manifestations and complications, especially in endemic regions.

## 1. Introduction

Infectious thyroiditis is unexpected due to anatomical and physiological characteristics, such as being encapsulated, rich blood supply, widespread lymphatic drainage, and high iodine concentration. The primary organisms involved are Gram-positive bacteria, including Staphylococcus and Streptococcus species [1, 2].

Acute infectious thyroiditis resulting from brucellosis is an exceptionally rare manifestation, primarily caused by Brucella species [3]. The phenomenon of thyroid involvement in brucellosis was first reported by Pacheco in 1963, and there are only a few documented cases in the literature [1].

Brucellosis is a zoonotic infection that endemic in many regions worldwide, particularly in areas with significant livestock populations. Humans can become infected through direct contact with infected animals or consumption of unpasteurized dairy products. The disease typically presents with systemic symptoms, such as fever, malaise, and fatigue, but its complications can affect multiple organ systems, including the musculoskeletal, cardiovascular, and reproductive systems [1, 3].

The pathophysiology behind Brucella-induced thyroiditis is not fully understood. It is hypothesized that the bacterium can invade the thyroid gland through the blood stream, leading to inflammation and subsequent clinical symptoms, such as neck pain, dysphagia, and systemic signs of infection [3, 4]. Diagnosis often relies on serological tests, with blood cultures serving as the gold standard, albeit with limited sensitivity in chronic cases [1, 3].

Early recognition and appropriate management of Brucella thyroiditis are crucial to prevent severe complications and improve patient outcomes [5]. This case report presents a case of acute granulomatous thyroiditis occurring in the context of brucellosis. It aims to highlight the importance of considering brucellosis in patients presenting with thyroiditis, particularly in endemic regions, thereby contributing to the growing body of literature on this rare but significant clinical entity.

## 2. Case Report

**Figure 1:**
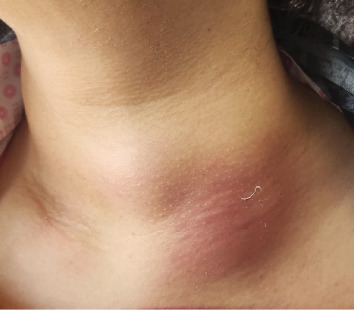
Unilateral erythema on the patients' neck on the first day of admission.

A 47-year-old female residing in a rural district was admitted to the emergency department on her family physician's recommendation to rule out anaplastic thyroid carcinoma. She complained of acute, progressive painful neck swelling with erythema and warmth, that had developed over the past 14 days (Figure 1).

The patient had been well until 8 months prior when she experienced notable pain in her left elbow and limited range of motion. After consulting an orthopedist, she recovered with painkillers. Given her occupation as a cattle farmer and her son's concurrent brucellosis infection, she was tested for brucellosis at that time; however, Wright and 2-mercaptoethanol (2-ME) titers were negative. For the past 2 months, she had reported significant fatigue and weakness.

Although she did not experience fever, she had episodes of chills and sweating in recent weeks and reported weight loss. Vital signs on arrival indicated an axillary temperature of 37.2°C, blood pressure of 110/70 mmHg, a pulse rate of 112 bpm, and absence of respiratory distress.

On neck examination, a large, unilateral, warm, tender, and fluctuant mass was identified alongside three palpable lymph nodes in the anterior cervical chain. She did not report difficulty breathing or swallowing. Other examinations showed no significant findings, such as tracheal deviation or hepatosplenomegaly. The patient had no notable past medical history, took no medications, and worked on her family farm.

Initial laboratory results revealed:

• Hemoglobin: 11.4 g/dL (normal values: 12.3–15.3).
• White blood cells: 6.6 × 10^3^/µL (normal values: 4.4–11 × 10^3^), 34% lymphocytes and 58% neutrophils.
• C-reactive protein (CRP): 58 mg/L (normal values: upto 7).
• Erythrocyte sedimentation rate (ESR): 73 mm/h (normal values: upto 20).
• Albumin: 3.3 g/dL (normal values: 3.9–4.9).
• Serum triiodothyronine (T3): 125 ng/dL (normal values: 80–190).
• Total serum thyroxine (T4): 11.4 µg/dL (normal values: 5–13).
• Thyroid-stimulating hormone (TSH): 0.026 mIU/L (normal values: 0.5–5.5).
• Negative results for HIV, HBV, and HCV.

The blood cultures remained negative throughout the 3-day incubation period.

Following admission, ultrasonography revealed right and left thyroid lobes measuring 36 mm × 14 mm × 17 mm and 18 mm × 13 mm × 23 mm with volumes of 4.9 cc and 4.1 cc, respectively. A heterogeneous hypoechoic solid/cystic region measuring 162 cc was noted adjacent to the left thyroid lobe, extending into the lateral neck and superior mediastinum, with a connection to the posterior aspect of the left lobe—suggestive of either an abscess or malignant lesion, such as anaplastic carcinoma. The radiologist also noted several round hypoechoic lymph nodes on the left side of the neck.

**Figure 2:**
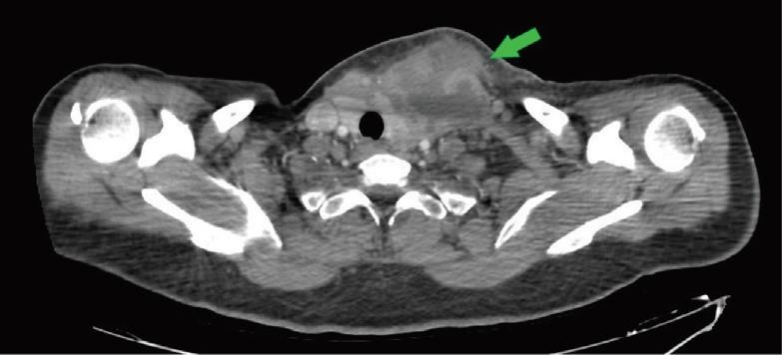
CT scan shows a fluid collection with a thick, irregular wall in the lower cervical region. Tracheal deviation is seen.

**Figure 3:**
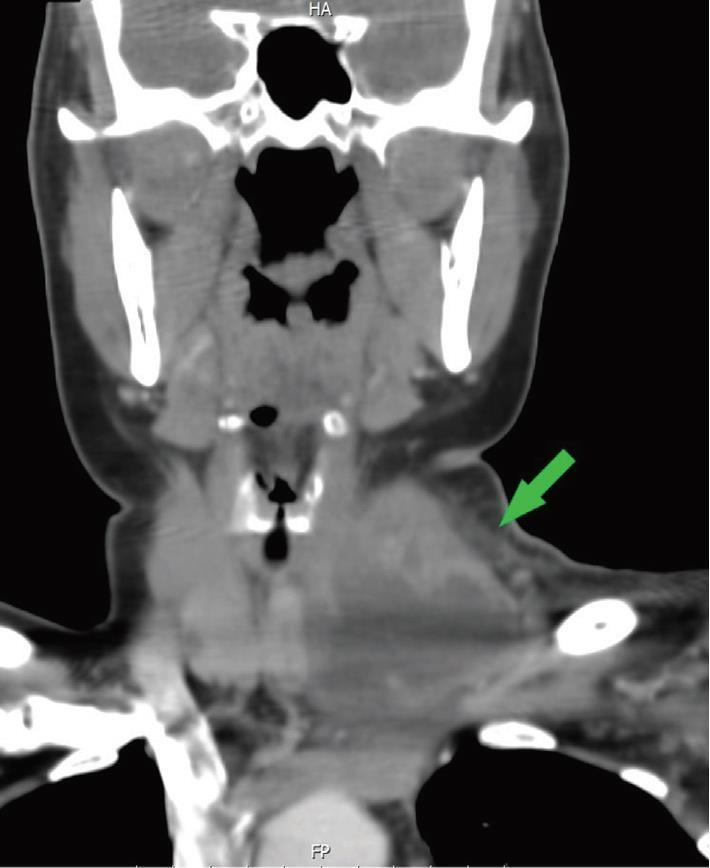
CT scan shows a coronal view of the abscess.

A cervical CT scan with contrast revealed a fluid collection with a thick irregular wall and peripheral fat stranding measuring 40 mm (anterior–posterior) × 61 mm (right to left) × 37 mm (superior–inferior) in the anterolateral region of the left thyroid lobe in the lower cervical region. This collection appeared connected to that in the left lobe and exerted mild pressure on the trachea (Figures 2 and 3). Thrombosis was also observed in the proximal part of the left jugular vein behind the abscess. The radiologist concluded that “suppurative thyroiditis is the primary diagnosis based on these findings.”

Considering high ESR and CRP levels alongside potential suppurative thyroid abscess indications, meropenem and linezolid were administered at appropriate doses prior to surgical intervention for abscess drainage. Three days later, she was transferred to the operating room for drainage of the abscess. The surgeon reported the drainage of a large suppurative abscess and performed debridement of necrotic tissue from the sternocleidomastoid muscle, while noting that the direct examination of the thyroid was normal.

Before surgery, due to previous history of brucellosis in the patient's son and the patient's contact with sheep, wright and 2-ME was sent which results were determined after surgery that positive with titer of 1/640 and 1/320, respectively; however, Brucellosis real time polymerase chain reaction (PCR) of blood was negative.

**Figure 4:**
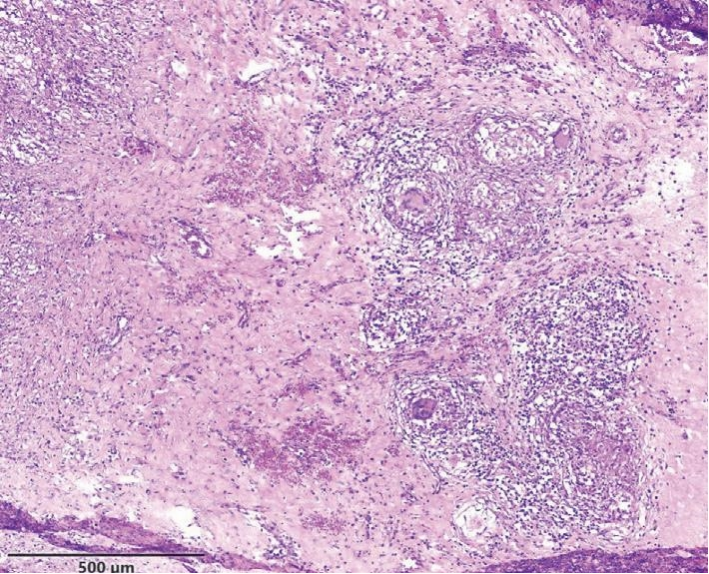
Histologic sections show marked inflammation with granulomas containing multinucleated giant cells and epithelioid histiocytes without caseation necrosis. Areas of fibrosis are also seen. The findings are diagnostic of subacute granulomatous thyroiditis.

The drained fluid from the abscess showed numerous white blood cells with predominant neutrophils; cultures remained negative after 72 h. Histopathologic evaluation showed granulomatous inflammation with epithelioid macrophages and multinucleated giant cells, features that can be seen in tuberculosis (TB). Ziehl–Neelsen staining, however, did not reveal any acid-fast bacilli (Figure 4)

Subsequently, oral doxycycline (100 mg twice daily) and oral rifampin (300 mg twice daily) were initiated, while previous antibiotics were discontinued. Apixaban was prescribed for long-term use due to jugular vein thrombosis. Anti-brucellosis therapy was continued for 6 weeks.

The culture of the fluid abscess became positive for *B.melitensis* after 2 weeks, identified by conventional biochemical tests and monospecific anti-*B melitensis* antiserum, which confirming the diagnosis of brucellosis. She was discharged from the hospital after 10 days of admission with a normal thyroid examination and in good general condition.

At the end of the course of treatment and also 8-months follow-up, the patient was clinically cured, and thyroid function tests and imaging, including ultrasound, were normal.

## 3. Discussion

Acute bacterial thyroiditis is a rare phenomenon, which is attributed to natural defense mechanisms of the thyroid gland. These include its anatomical position, encapsulated structure, lack of direct communication with adjacent tissues, high iodine content, rich vascular supply, and extensive lymphatic drainage in the neck [5]. In this study we described a rare case of thyroiditis induced by Brucella species and then abscess formation due to delayed treatment.

Cvetkova et al. [1] shared a case of brucellosis that had neck pain and swelling on the second day of hospitalization after treatment was started. Treatment was successful without drainage [1]. A review of the literature in Turkey involving 1028 patients with brucellosis found only one case with thyroid involvement as a complication [6]. Abscess formation may be expected if primary brucellosis infection is left untreated [7]. Akdemir et al. [5], documented a case of postpartum neck abscess with positive fluid culture for *B.melitensis* that underwent drainage; treatment was initiated afterward.

In previously reported cases of Brucella thyroiditis, thyroid function tests typically remained within normal ranges [1, 4]; however, deviations can occur, such as suppressed TSH and elevated T4 levels [5, 8]. In our case, upon admission, thyroid tests indicated suppressed TSH, while total T4 levels were normal. It is plausible that the patient experienced a phase of thyrotoxicosis weeks prior to hospitalization, resulting in normal T4 levels but still suppressed TSH at the time of examination.

In our case, ultrasound findings were distinct from most reported Brucella-related thyroid abscesses, which typically present with smaller, localized hypoechoic or anechoic collections within the thyroid parenchyma. For instance, Akdemir et al. [5] reported the abscess was confined to the thyroid with significant enlargement, but lacked mediastinal extension, while another study described a smaller, irregular hypoechoic nodule without cystic components or nodal involvement [9]. Similarly, two published reports demonstrated diffuse thyroid heterogeneity or focal hypoechoic areas but no large cystic-solid masses or mediastinal spread [1, 3]. The extensive lesion in our case, with its atypical size and extracapsular invasion, overlaps more with aggressive pathologies, like anaplastic carcinoma. It highlights the diagnostic challenge in distinguishing Brucella-induced abscesses from malignancy, particularly given the rarity of such large, infiltrative abscesses in brucellosis-related thyroiditis.

Serologic testing is the most commonly used method of diagnosing brucellosis; however, it has some limitation. In endemic regions, these tests may show a broad range of susceptibility and lack specificity. PCR testing has proven to be one of the most useful assays for diagnosing human brucellosis, demonstrating 91.9% sensitivity and 95.4% specificity in active cases. False-negative PCR results can arise from low organism counts below detection limits or from degradation of target DNA in samples. In our testing, the serology results were positive despite negative PCR findings. Notably, the PCR was performed 6 days after initiating antibiotic therapy. The observed discrepancy may be attributed to the progressive decline in target bacterial DNA load due to antibiotic treatment. It may lead to false-negative PCR results over time. This highlights the critical impact of testing timing relative to antibiotic administration on molecular assay sensitivity [10, 11].

The pathology report indicated granulomatous inflammation, necessitating the considering of TB as a differential diagnosis. Infectious sources for granulomatous thyroid disease are rare; however, there are documented cases of thyroiditis associated with TB, which is the most common infectious etiology for granulomatous thyroid disease [12]. The likelihood of TB infection was considered low given the patient's positive serologic tests for brucellosis, occupational exposure as a cattle farmer, and a recent confirmed case of brucellosis in a close family member. In our case, TB was ruled out by low levels of ADA in abscess fluid and negative Ziehl–Neelsen staining for TB, as well as, the abovementioned clinical and epidemiological factors.

The IGRA test was negative in our patient, while a negative IGRA does not definitively exclude active TB; it indicates no prior sensitization to *Mycobacterium tuberculosis*. The reliability of a negative IGRA is reduced if performed within 8 weeks of exposure or in immunocompromised individuals [13]. Given that our patient has been symptomatic for several months and is immunocompetent, the negative IGRA result carries a higher negative predictive value for excluding active TB.

Importantly, brucellosis has also been reported as a rare infectious cause of granulomatous inflammation in various organs. Yalcin et al. [14] noted brucellosis as a rare cause of granulomatous hepatitis, with *Brucella abortus* being the most common species responsible for hepatic granulomas.

During the initial visit, the physician considered a diagnosis of thyroid cancer due to the rapid progression of a neck mass, accompanied by suppressed TSH levels resembling malignant patterns. Anaplastic thyroid carcinoma can present as a rapidly enlarging anterior neck mass with accompanying symptoms, such as noticeable lymphadenopathy and neck pain—symptoms present in our case. However, other common symptoms like dysphagia, voice changes or hoarseness, and stridor were absent [15]. However, clinical response and culture reports confirmed the primary diagnosis; distinguishing between thyroiditis and malignancies presents clinical challenges [16, 17].

The CT scan revealed internal jugular vein thrombosis attributable to local infection, which is one of the main etiologies of this condition [18].

In conclusionꓹ it is advised to consider brucellosis as a differential diagnosis in patients with thyroid masses in endemic areas. Diagnosing brucellosis needs high suspicion. Patients with brucellosis may suffer from long-term complications that often go undiagnosed due to the variability and nonspecificity of symptoms [8]. This case report highlights the variation of organ involvement during the *B. melitensis* infection course and it may help to better understand the unreported aspects of this disease.

## Data Availability

Data supporting this study are available from the corresponding author upon request, but are not publicly accessible due to privacy/ethical restrictions.
